# Global cargo gravitation model: airports matter for forecasts

**DOI:** 10.1007/s10368-021-00525-2

**Published:** 2021-11-27

**Authors:** Fabian Baier, Peter Berster, Marc Gelhausen

**Affiliations:** German Aerospace Center (Deutsches Zentrum für Luft- und Raumfahrt e.V.), Köln, Germany

**Keywords:** PPML gravity, Air cargo, Airport fixed effects, Aviation, Traffic, Forecasts, C53, F17, F63, F69, R41

## Abstract

The reliability of forecast models in the aviation sector is an important factor for industry and policy makers likewise. Expanding airports and fleets usually is a cost and time intensive process, and in order to maintain efficient market behavior, accurate anticipation of future demand and structural changes is attempted. We present a new quantitative approach to air cargo forecasts utilizing global airport-dyadic ICAO CASS data in general linearized airport fixed effects gravity models. While the strong explanatory power of our time invariant constant model has its natural difficulties predicting a variety of smaller indicators from previous models found in literature, we achieve very good results for selected time variant variables as gross domestic product per capita or kerosene prices. This makes our model a perfect tool for forecast simulations: extrapolating general economic forecast data provided by IHS Markit yield similar results to Boeing cargo forecasts (2020), with a slight decrease in the long run. Additionally, we do not need to split or control our sample in regional groups due to airport fixed effects, which makes the model on the other hand suitable for country- and airport level forecasts as well. The utilization of a large unique bilateral freight data set also helps answering classical gravity model questions in aviation: we track the distance effect to a matter of sample selection, finding no significant interaction following state of the art gravity econometrics.

## Introduction

Global economic interaction has vastly increased over the past decades, leading to exponential growth paths of cross-border economic activities. More detailed and specialized supply chains in production as well as consumption patterns across countries lead to an increase in complexity and thus require more sophisticated logistic structures. Airfreight makes up for a rather small amount in terms of both ton-kilometers and tons – only around half a percent (Statista 2021a) in comparison to the 8.9 billion tons of goods transported by rails (International Union of Railways 2020) and about 11 billion tons by sea (Statista 2021b) in 2019. When looking at the value of transported goods however, air transport is the preferred choice with about 40 percent of total value of transported goods, including special industrial freight and high-quality consumer goods and postal service. The annual growth rate of air freight continues in exceeding the total growth rate of global freight, attracting special attention from both researchers and businesses.

In order to maintain functional and efficient logistic structures it is crucial to plan and prepare for future demand for transportation, whereby this study targets specifically the aviation sector. We develop a global air freight demand model which meets the requirement to be utilized as forecast tool on country- and airport level, supporting a more found derivation of fleet- and airport (hub) development. A disruptive factor in the quantitative approach remains unprecise trucking networks processing airfreight from and to airports, i.e. it is difficult to automatize a clear-cut catchment area of airports regarding freight. Our findings however help limiting disruptions and give a roadmap on how to approach regional airport-economy.

While we find a broad variety of approaches to model national- and regional airfreight demand in the literature, recent global air freight models (Airbus 2019, Boeing 2020, Karp Lufthansa Consulting 2020, IATA 2020, 2021, ICAO 2021) are similar in their methodology, forecasting annual average air freight growth between 3.5 % and 4.2 % up to the year 2045. The underlying paper aims to develop the intuitive gravity models (Shepherd 2017) being currently prevalent in literature by utilizing airport fixed effects in order to control for the vast number of unobservable factors. Following this approach leads to highly adjusted gross domestic product (GDP) coefficients, thus making it possible to formulate more precise elasticities for air cargo demand with regards to GDP per capita. Controlling for (micro) unobservable factors on regional level we find (opposing to previous studies) that the annual average oil price does not have a significant impact on air freight; this effect should rather be credited to airport- and/or country-specific time invariant characteristics. In contrast, the annual average kerosene price has a much higher explanatory power than oil prices. Because of correlation issues with GDP per capita, we estimate both coefficients in separate models and apply their calculated impact in the forecast. We find that the kerosene price captures annual time variances and shocks very well and thus is a good indicator for annual fixed effects, while a pure GDP per capita based forecast model with time fixed effects has a good long-run fit.

The paper is structured as follows: Chapter 2 looks at current literature with regards to air freight models and forecasts. Chapter 3 describes data and methodology and estimates a variety of models. In Chapter 4 we apply our results to generate several forecast scenarios and discuss the pros and cons of our approach. In that context we will discuss how to employ our findings for regional airport analyses. The paper closes with a brief conclusion and some recommendations for future research in Chapter 5.

## Literature review

There has been already quite some research on air freight demand modelling and forecasting, however, much less compared to passenger demand which typically gets more attention (Gelhausen et al. 2019 for a brief overview). If we take a look at the existing literature, there are different approaches to air freight modelling, ranging from the classical gravity model, to time series models such as error correction or ARIMAX models and finally models such as neural networks and system dynamics. There tends to be a far wider range of model approaches compared to passenger demand modelling, where in many cases the gravity approach is applied. Hereinafter, we give a brief overview over the various model approaches.

Alexander and Merkert (2021) estimate a gravity model for international air freight in the US in the post-global financial crisis era. Main drivers of air freight are transport cost, competition from sea freight and consumer spending patterns regarding perishable, low value and high value goods. Hamal (2011) develops a double logarithmic linear single equation model, which is technically a gravity model, to model air freight at Australian airports and establishes a forecast up to 2030. Key variables that he identifies as drivers of air freight are population, income, prices and exchange rates.

Wadud (2013) employs an approach that integrates passenger and air freight demand modelling. Based on time series data, he uses a seemingly unrelated regression (SUR) model to model passenger and air freight demand at Shahjalal International Airport at Dhaka in Bangladesh. Main drivers of air freight are gross domestic product (GDP), national price levels and the oil price. Kupfer et al. (2017) develop an error correction model for global air freight development. Important determinants of air freight development are amongst others merchandise trade and the share of manufacturers, air freight yields as well as the oil price. Different scenarios are created with regard to these drivers to forecast global air freight in 2023. Gudmundsson et al. (2021) constructs an ARIMAX model to forecast the COVID-19 recovery of global air passenger and air freight markets. GDP is used as an explanatory variable in the ARIMAX setup. Their key findings are that air freight recovery is faster and less uncertain compared to the passenger market and will take on average about 2.2 years. Recovery of the air freight market is not as steep as that of the passenger market, because the recession was not as distinctive. Lakew and Tok (2015) develop a model based on the reduced-form equation that treats price endogenously (Brueckner 1985) based on a seven-year panel. Various socio-economic factors are included, like population, wages, share of service-related and manufacturing-related employment. Finally, they present a 2010 to 2040 forecast for California.

Further approaches include a neural network-based approach of Chen et al. (2012) to forecast air freight demand from Japan to Taiwan, a system dynamics approach of Suryani et al. (2012) including optimistic and pessimistic scenarios for assessing airport infrastructure, especially terminal capacity, to handle air freight and Chou et al. (2011) with a fuzzy regression approach to reduce forecast uncertainty.

Reference industry long-term air freight forecasts are Airbus (2019), Boeing (2020), IATA (2020, 2021) and ICAO (2021). They forecast annual growth rates of between 3.5 % and 4.2 % up to the year 2045.

## Data, methodology and model estimation

As the literature review makes clear, there is a multitude of possible explanatory variables in an air freight demand model. Most notably we find GDP- and transport-related variables like, e.g., oil or jet fuel prices. Our goal is to develop a global air freight demand model which is based upon the airport pair level, i.e. air freight between any two airports around the globe. While it is not our intention to exactly forecast air freight demand for each airport pair, we aim to improve forecast accuracy on a higher level, e.g., airport region totals, as we will see further below. While there are data availability limitations on a global level and strong correlations between different variables, we have to make a choice as to which variables to include in our model. We therefore have focused on GDP-, distance- and transport-related variables as well as airport-specific factors so that we may have incorporated the better part of influencing factors in our approach.

### Data

We use annual aggregated ICAO CASS air freight data on airport to airport level (origin, destination) from 2010 – 2019 in ton-kilometers, the CEPII gravity_dist databank for bilateral constants (cultural, historical and physical distance), World Bank (GDP, GDP per capita and oil price in USD^1^) and OECD (FDI and trade in USD) data for economic indicators, and kerosene-type jet fuel prices by the U. S. Energy Information Administration (in USD). For our forecasts, we use OECD GDP per capita data for aggregated global forecasts and IHS Markit data for country- and region-specific forecasts.

Bilateral non-available freight positions are assigned to zero and make (together with natural zeroes) 37,432 data points, an important information being able to include in poisson-based estimations (in contrast to ordinary least squared which drops zeroes in order to logarithmize).^2^ This carries the assumption that non-reported freight positions are either not existent or too small to have an effect. Thus, our dataset contains 1,038,933 origin-destination annually aggregated freight data points on airport level across 10 years, with a total calculated data intersection of 1,002,201 observations. However, not all airports and countries globally are covered, due to the nature of the data collection method by ICAO CASS participants. ICAO CASS data covers 1233 origin- and 3186 destination airports. A major fraction of African and middle-eastern countries is missing, as well as several small island states and states in central- and Latin America, which is important when applying results for forecast generation and interpretation. A split in income level of countries and regions and continents as proposed by Boeing / ICAO research is expected to be covered on a more precise level due to airport fixed effects and GDP per capita. Regarding forecast precision an option is to swap regional and continental splits, the dimension of effects should however be quite small due to fixed effects. On the other hand, as we have a coefficient for each individual airport, we can in theory give airport-specific forecasts.^3^ Separating the dataset into regions and continents can however remain an interesting robustness check (this is not yet part of this research).

### Methodology

Empirical estimation of the global air freight network is based upon the principles of Newton’s law of gravitation, commonly adapted to economics starting 1962 with Tinbergen, who finds that bilateral trade can be explained with variables of distance between trading partners and their economic size already to a good extent. Gravity models have been adapted by researchers over the decades and apply them to quantitative research in cross-border trade & investment, but as well in migration- and traffic research. While gravity models are in some fields struggling with their microfoundation, we find a solid theoretical framework adaptable to airfreight by Anderson and Van Wincoop (2003) with a caveat to transportation cost.^4^ A special point of attention is put on multilateral resistance terms, describing the matter that a change in flow of goods between airport A and B indirectly affects the flow of goods between all other airports; i. e. if a certain amount of freight is re-assigned to another transport route, it is automatically negatively affecting the route it has been transported so far. This can be achieved by adding a respective set of fixed effects to the model (Santos Silva and Tenreyro 2006, 2010; Baldwin and Taglioni 2007; Fally 2015; Larch et al. 2019). In aviation economy, fixed effects gravity to model multilateral resistance is not yet broadly adapted, only a handful of passenger studies work with panel fixed effects models (Piermartini and Rousová 2013; Cristea et al. 2015; Zhang et al. 2018). According to the authors’ knowledge, the underlying paper is the first one to utilize airport fixed effects instead of country fixed effects in order to picture a more detailed traffic network. We reckon two reasons for this: First, the data situation is not easily accessible. Second, a vast amount of control variables (one for each origin- and destination airport) combined with observations lead to technical estimation challenges for which programming solutions have not been available until just recently. Put simply, the vast amount of observations and fixed effects have not been able to be processed with conventional estimators so far (for a discussion and mathematical analyses, see Stammann et al. 2016). The advantage of a more detailed fixed effects model is to counter disturbance to our control variables, which are therefore estimated more precisely. Increasing the number of control variables however significantly rises the potential of overfitting – in that case the constants swallow all explanatory power and the model becomes inadequate for forecasting. Using airport-pair fixed effects would thus lead to a collapse of our model, which stands in contrast with country-pair fixed effects trade models occasionally found in trade literature.

When it comes to the choice of regressor in the presence of heteroscedasticity and values of zeroes in the dataset, the poisson pseudo maximum likelihood (ppml) estimator developed by Santos Silva and Tenreyro (2006) yields the most consistent results as well with increasing number of observations (Kareem et al. 2016; Baier 2020). Heteroscedasticity is found to be a common problem in fixed effects gravity estimations which we thereby avoid in an early stage (Silva and Tenreyro^ 2011^). As mentioned in the passage above, the ppml estimator by Santos Silva is limited in its use, thus we work with generalized linear model estimation approximating ppml (Stammann 2018). For ease, we illustrate the ppml estimation problem according to Larch et al. (2019) with coefficients β, fixed effects v and error term ε as follows:

$${freight}_{odt}=\mathrm{exp}\left[{\beta }_{1}\mathrm{ln}\left({dist}_{od}\right)+{\upbeta }_{2}\mathrm{ln}\left(gdpcapit{a}_{ot}\right)+{\upbeta }_{3}\mathrm{ln}\left(gdpcapit{a}_{dt}\right)+{\beta }_{4}fue{l}_{t}+{\beta }_{5}culturedis{t}_{od}+{\beta }_{6}conti{g}_{od}+{v}_{o}+{v}_{d}+{v}_{t}\right]+{\varepsilon }_{odt}$$

whereby the dependent variable denotes the freight flow from origin to destination airport in year t. Distance is measured in physical distance as well as cultural distance (common language, common historic and colonial ties) between countries the airports are located in, and the GDPs per capita of respective countries are used. Contiguity as measure whether countries share common borders is controlled for, and time-variant annual average jet fuel price serves as shock-indicator alternative to yearly fixed effects.

The approach to use airport specific data with country specific controls is not commonly used in gravity models due to data limitation. While we could use distance data between airports and approximate regional GDP per capita for airport regions or specific cities, this approach is likely to disturb our findings to some extent as national industries and airports are linked within complex logistic systems, which are: a) unlikely to be radial and b) cover as well broad regions which have no direct airport access. The distance between airports within countries matters especially for heterogeneous countries like Russia and China (see Wang et al. 2018) for which we average over GDP per capita. National truck and railroad networks have to be considered for specific regional forecasts, while our model map international and global freight movements and thus can be applied to forecast future global demand.

### Model estimation

It is noted that several test runs were performed checking the explanatory power of foreign direct investment and trade variables to our models, finding either no significant impact or endogeneity on the amount of air freight. Testing with both GDP per capita and GDP were done and we decided for the latter due to a better fit of the model in terms of pseudo-R^2^ and as well more robust results across our testing series. Opposing to previous studies, oil prices were found to have no significant effect in the presence of airport fixed effects. Kerosene prices are found to have a strong impact however even in the fixed effects model. The issue here is that we find a high correlation to our GDP based variables and cannot test both in the same model and use the results for forecast generation. Perfect collinearity with annual time fixed effects is observed as well due to the nature of the variables (kerosene price as global average on annual basis). Thus, the forecast solely bases on GDP per capita growth and fuel price is only used to control for annual shocks in our sample period. Forecasting (economic) shocks is a contradiction in itself as they have the nature to occur unexpected – we can however apply a fuel effect to future air traffic by assuming constant price development with decreasing airplane fuel demand. This shall however only be discussed in the framework of the underlying paper, and not applied quantitatively.

We test several data samples to view the difference across the treatments “all data” (1), “without zeroes” (2) and “large flow only” (3) in a first step and display our results in Table 1. We find that the information to which airports less freight or no freight is transported is important to include in our model, which is as well in line with econometric theory (Kareem et al. 2016). While model (3) only considers large flows and can therefore rather be seen as macro-founded data source, we see the bias especially with regards to the distance effect when using the complete data set, including all available (also very small) flows as in model (1).

**Table 1:** Estimation results for total data (1), without zeroes (2) and only above 50k ton-km of freight transported(3; with n = observations and l = fixed effects [origin, destination, time]

	**(1)**	**(2)**	**(3)**
log(dist)	-0.009 (0.013)	-0.013 (0.013)	-0.310*** (0.015)
log(gdp_cap_o)	0.768*** (0.130)	0.770*** (0.130)	0.607*** (0.129)
log(gdp_cap_d)	0.525*** (0.111)	0.522*** (0.112)	0.510*** (0.113)
contig	-0.521 *** (0.052)	-0.527*** (0.052)	-0.566*** (0.048)
comlang_off	0.303*** (0.022)	0.301*** (0.022)	0.264*** (0.022)
colony	0.665*** (0.024)	0.662*** (0.024)	0.482*** (0.026)
comcol	0.176*** (0.042)	0.173*** (0.042)	-0.035 (0.041)
n	1002201	966290	160861
l	[1233, 3186, 10]	[1233, 3186, 10]	[544, 776, 10]
pseudo-R^2^	0,7502	0,7487	0,6632

Standard errors in parentheses

*** p<0.01, ** p<0.05, * p<0.1

Model (1) is therefore selected as our model of choice for GDP per capita based estimation.

The role of distance in gravity aviation is quite disputed in literature. While most sources especially in passenger aviation find negative signs, it is sometimes found as well to have no impact. We can observe that this might be a sample selection problem, as we in fact find a negative and highly significant sign if only considering large airports (top 15 %). In the picture that most publicly available data covers mainly large airports and movement this should be discussed in future research. In the framework of the underlying paper, distance is however treated as pure control variable.

In our model of choice, the GDP per capita coefficients for both origin- and destination country (“gdp_cap_o” and “gdp_cap_d, respectively”) are highly significant, with origin being slightly more intense. For each percentage point of growth in GDP per capita, a country will therefore send 0.77 % and receive 0.53 % more freight, an interaction important to consider in a global context; therefore, an individual country’s freight development depends on [the differential of own GDP per capita], but as well on [the differential of global average GDP per capita]. Applying the results to global GDP per capita forecasts from OECD (2018), we find that global air freight will approximately increase +4.4 % from 2018 to 2030, and +3.1 % from 2030 to 2060, on annual average basis respectively, and thus in range of previous studies.^5^

The coefficient of our contiguity dummy (“contig”) shows a negative sign, stating that countries which share a common border are significantly less likely to use air transport systems for their goods exchange, which we interpret that it is more likely to use alternative transportation methods (truck, train or inland navigation) due to speed and cost advantage, whereby the caveat for larger countries applies (Wang et al. 2018). The coefficients for our cultural distance variables whether both countries speak the same official language (“comlang_off”), have colonial ties (“colony”) or are common colonizers (“comcol”) all show positive significant signs and thus are in line with gravity literature (for a broad overview, see Baier 2020). Those variables are merely used as bilateral time invariant control variables and thus excluded for the forecast. The rows n, l and pseudo R^2^ in Table 1 show the number of observations, the number of included fixed effects for [origin, destination, time] and the calculated McFadden pseudo-R^2^.

While dyadic fixed effects models are commonly utilized in national gravity models (trade, FDI, migration etc.), we face a mass problem when considering airports, as we have up to 4.500 potential origin- as well as destination airports, leading to millions of fixed effects and thus almost a pure constant model. In order to visualize the difference to airport FE, we attach Table 4 in the appendix. A set of 181.821 dyadic airport fixed effects lead to a pseudo R^2^ of 0.97; countrypair- specific characteristics as distance, contiguity etc. are dropped due to the nature of dyadic fixed effects. GDPs remain highly significant and with slightly higher coefficients than in sole airport fixed effects. It shall be noted as well that model (7) satisfies the multilateral resistance requirements by the Anderson and Van Wincoop (2003) micro-foundation. The forecasting power of (close to) constant models is however weaker as we can not track potential changes in variables as well (with the exception of GDPs). We thus decide to stick with model (1) as our model of choice. This does not contradict with Shepherd (2017), who discusses both country- and dyadic fixed effects models both as legit, even though country fixed effects models lack to control for multilateral resistance.

Table 2 shows the estimation results replacing annual fixed effects for jet fuel prices (5); Adding GDP per capita to the fuel model (6) the effect gets swallowed due to negative correlation between variables. In comparison, model (4) shows GDP per capita results without annual fixed effects and yields perceptible higher magnitudes than in model (1) with annual fixed effects, indicating that annual fixed effects smooth out economic shocks to air freight not directly linked to GDP based variables, making it thus necessary to control with a time tracker in order to get undistorted values for the GDP per capita coefficients.

**Table 2:** Estimation with fuel prices as shock indicator instead of time FE

	(4)	(5)	(6)
log(dist)	-0.008	-0.008 (0.013)	-0.008 (0.013)
log(gdp_cap_o)	1.002*** (0.101)		1.025*** (0.107)
log(gdp_cap_d)	0.678*** (0.092)		0.695*** (0.097)
fuel		-0.162*** (0.021)	0.018 (0.025)
contig	-0.520*** (0.052)	-0.518*** (0.0518)	-0.520*** (0.052)
comlang_off	0.304*** (0.022)	0.303*** (0.022)	0.304*** (0.022)
colony	0.665*** (0.024)	0.665*** (0.024)	0.665*** (0.024)
comcol	0.177*** (0.042)	0.179*** (0.042)	0.177*** (0.041)
n	1002201	1002201	1002201
l	[1233, 3186]	[1233, 3186]	[1233, 3186]
pseudo-R^2^	0,7499	0,7484	0,7499

Standard errors in parentheses

*** p<0.01, ** p<0.05, * p<0.1

Concluding we find robust results in model (1) for national GDP per capita coefficients in an airport fixed effects gravity model utilizing dyadic time invariant (physical and cultural) distance variables. Time fixed effects correct for shocks in the training period which would otherwise lead to an overshooting GDP per capita effect in our forecast. Fuel prices negatively correlated with GDP per capita in our sample 2010 to 2019 can be adjusted to explain shocks in the training period. Their application in forecasts is however limited due to the per definition unpredictable nature of economic shocks and is rather useful for scenario building.

## Forecast application

### Global and national forecast

An efficient way to generate forecasts in the aviation sector is applying basic economic forecast data such as GDP (per capita) on respective demand elasticities in regression models (Gelhausen et al. 2018). In literature we find that data of two providing sources are mainly used for such: OECD and IHS Markit. We already did a quick-check with global OECD data in chapter 3, being roughly in line with literature. Due to detailed country level data and for better compatibility with the DLR passenger flight model (Gelhausen et al. 2018) we continue using real GDP per capita growth forecasts in percent compared to the previous year respectively provided by IHS Markit (2017).

Global freight from origin- to destination country level can thus be modelled with the only time variant input variables of GDP per capita, as all other independent variables in our model (1) are time invariant constants. Besides generating global aggregated forecasts, country- and region-specific forecasts can be calculated.^6^ With regards to fuel cost we assume constant development for the future and thus exclude that effect in the framework of this chapter. It shall be noted that with our findings it is however possible to include fuel prices for scenario building, for example if we assume that jet fuel need will decrease by 1% annually due to increasing jet engine efficiency and/or new technologies such as electric or hydrogen engineering. Another possible use for short-term forecasts is noted as jet fuel prices can directly be observed and/or trends can be fixed. Due to very low kerosene prices in 2020 we thus expect an offsetting effect to the ongoing COVID-19 crisis, which we cannot yet evaluate by global air freight data.

Checking the fitness of our forecast data we use the years 2010 to 2019 as sample data and reverse an artificial forecast taking the 2019 cargo volume as base case. Growth paths are adjusted accordingly via fuel price in that time frame. Starting 2020 with the actual forecast we continue via time fixed effects control model as we do not want to rely on numerous additional uncertainties such as fuel price forecasts.

**Fig. 1:**
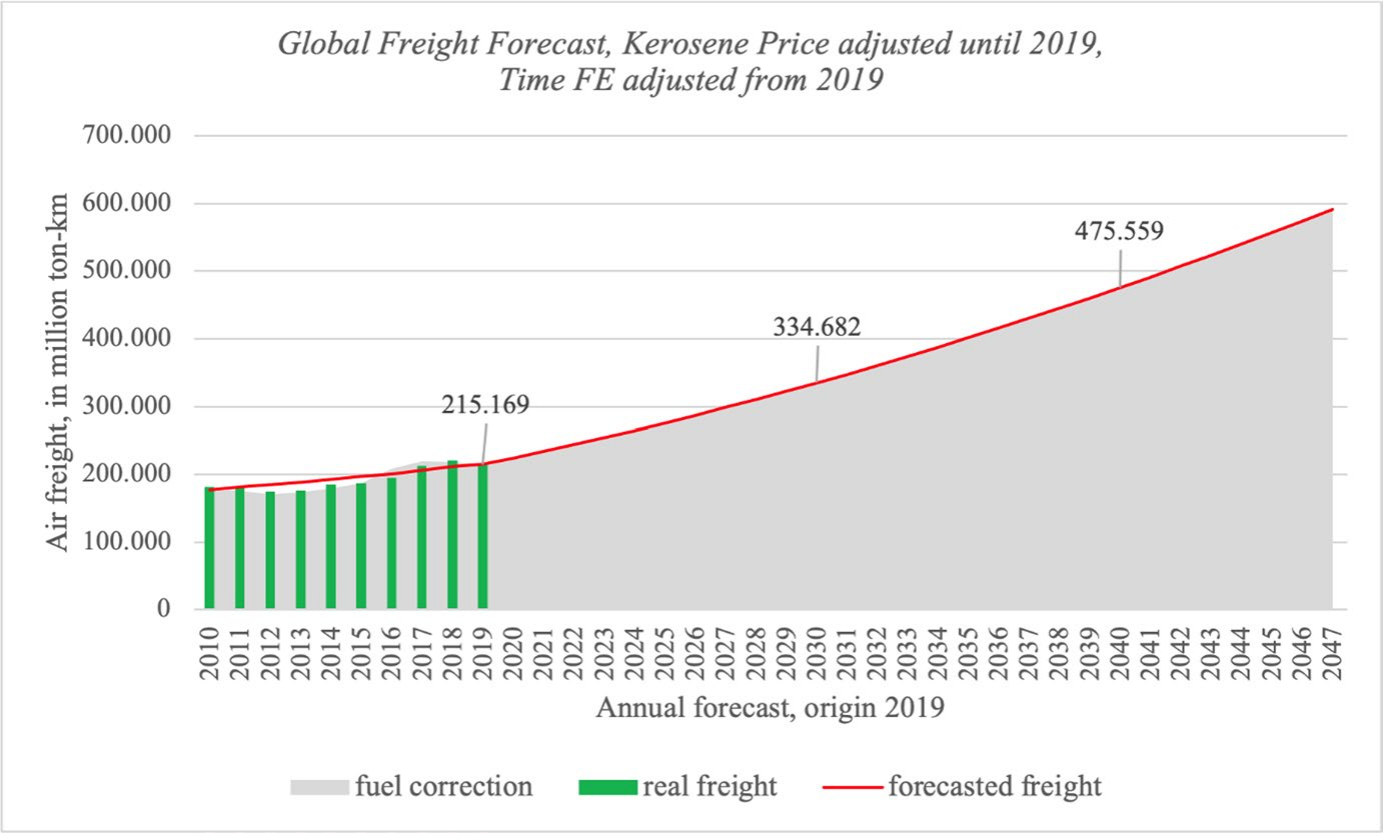
Global Freight Forecast, Kerosene Price adjusted until 2019, Time FE adjusted from 2019

Figure 1 thus shows the actual (green) and forecasted (red / grey) global air freight development in million ton-km (World Bank Data), whereby the red line represents forecasts generated with model (1) which we adjust 2010 to 2019 with Kerosene prices. The backward fit to 2010 is quite exact to the actual freight transported in that year, where 182.026 million ton-km air freight were observed (World Bank). Taking the base year 2019 we calculated 177.400 million ton-km air freight for that year, and 176.352 million ton-km if we additionally consider kerosene prices. For the year 2030 we forecast 334.682 million ton-km air freight assuming global average growth in GDP per capita of 3,2 % (IHS Markit). This leads to expected freight growth of in average 4,1 % per year, 2020–2030 and thus we are in line with Boeing (2020). Looking at the decade 2030–2040 our model predicts a decrease of the growth speed, where annual average growth will be 3,6 %. For 2040–2047 average growth will continue to decrease to 3,2 % per year in average. In sum, our forecast thus is slightly lower than the Boeing (2020) forecast which we track back to the different methodology we use, naming our airport fixed effects gravity with ppml regressors.

Looking at the explanatory power of our model to the testing period 2010–2019, we observe an overshooting of our time fixed effects model especially for the years 2012 and 2013 with too high estimation if not controlling for fuel price. In those years aviation underwent a small decline (instead of growth) which are explained by IATA as follows: “… the air cargo market contracted by 1.5%. The industry suffered a one-two punch. World trade declined sharply. And the goods that were traded shifted towards bulk commodities more suited for sea shipping,” (IATA 2013). Especially in those years we achieve a good fit to the actual data by controlling for the relatively high fuel prices. Including market shocks into the forecast model starting 2020 is however not trivial, as explained above, and thus mainly helps to identify drivers in the past and current / within year shocks. For longer time periods and decades our time fixed effects model proves to be very accurate on the other hand.

**Fig. 2:**
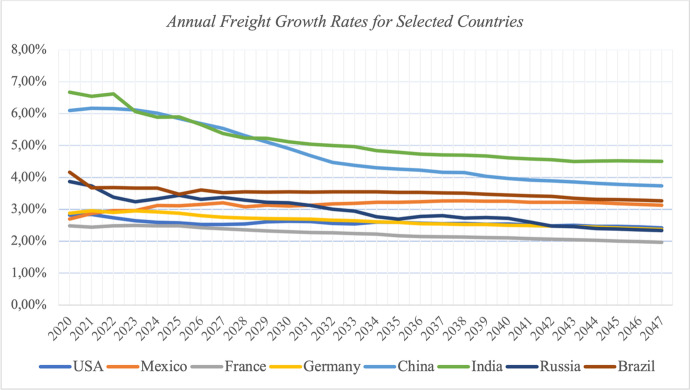
Annual Freight Growth Rates for Selected Countries

Figure 2 shows the annual air freight growth rate forecast from selected countries, whereby national GDP per capita (origin) is considered as push-factor for outward transportation and global GDP per capita (destination) as pull factor. Utilizing this forecast methodology, we can as well forecast freight traffic between regions (counties, states, unions, continents etc.) by averaging respective GDP per capita measurements for origin and destination, plus inserting absolute current freight data as starting basis for the forecast. For Fig. 2, we assume national interaction with the total rest of the world, finding annual growth rates above 5 % for China and India until 2030 and overall decrease in growth rates.

**Fig. 3:**
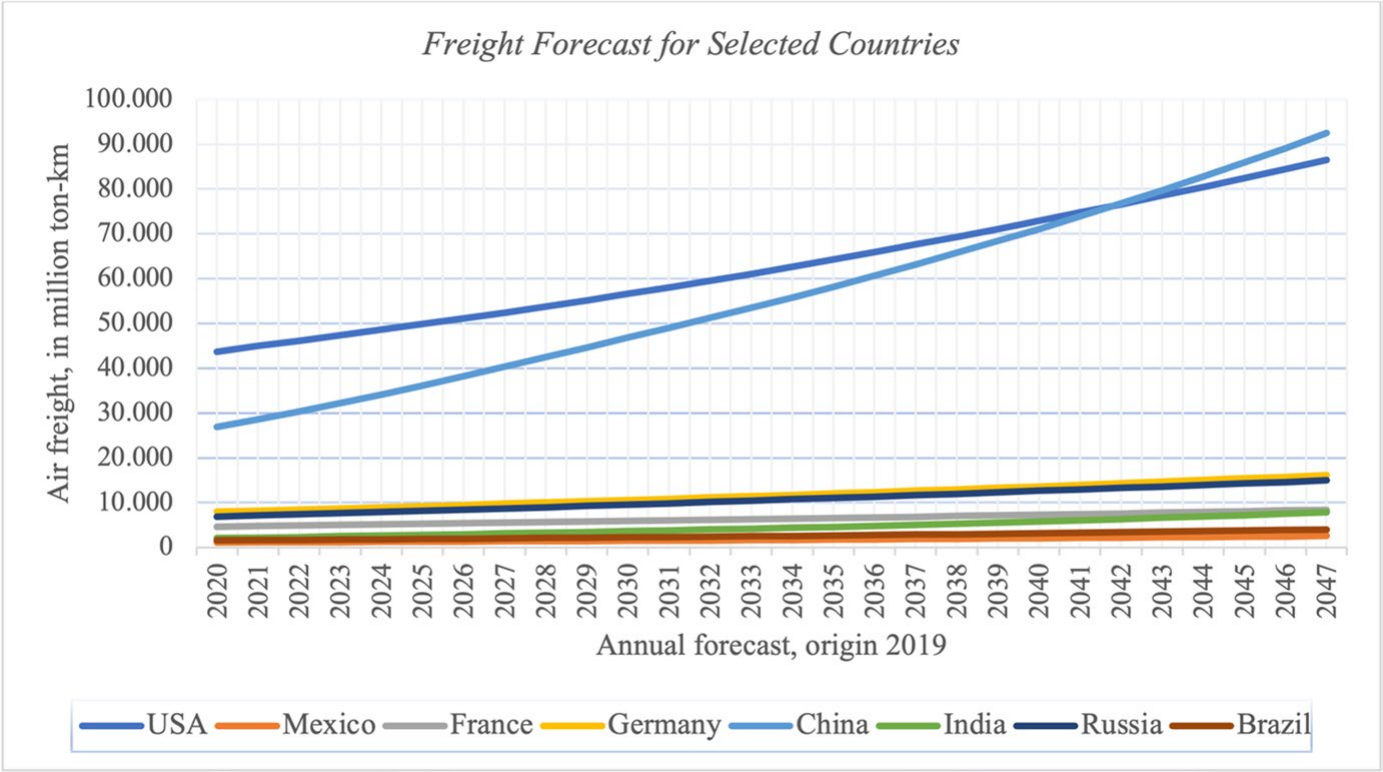
Freight Forecast for Selected Countries

Figure 3 applies the growth rates to 2019 base data, averaging annual air freight in million ton-km for the years 2020–2047. We observe that China is going to pass the USA in terms of mass transported by air in approximately 2042 and India is likely to catch up to about the level of France by mid of the century.

As announced, we as well want to consecrate a passage of this chapter to kerosene prices which are not quantitatively considered in above forecasts. While kerosene price estimations can be linked to oil price predictions provided by all major investment corporations (U.S. Energy Information Administration for an overview) the forecasts are inherently relevant for short time periods and highly sensitive to daily business and economic shocks (Frey et al. 2009). As our goal in this paper is to develop a reliable long-term forecast model, oil prices add unwanted volatility and thus are not optimal to include. They can however be considered for scenario building; Introducing model (5) to the assumption that due to increasing performance / backstop technologies to airplane engines fuel savings of additionally 1,0 % per year occur, this would lead to a global air freight traffic plus of 0,16 %.^7^ Opposing effects occur if increasing kerosene demand leads to mark-ups of fuel prices due to resource scarcity, which will then have an overall negative effect on global air freight volume.

Air traffic is expected to double within the next two decades, so that environmental bottlenecks need to be considered by industry and policy makers. Currently 3.5 % effective radiative forcing^8^ can be traced back to aviation (Richie 2020). While optimizing technologies like hydrogen and electric motorization lead to cleaner aviation, fleet evolution is likely to follow airplane retirement plans and economic considerations made by airlines. Thus, reducing emissions in the aviation sector takes time and may even increase in the short term due to air traffic growth. As a result, there may be increased environmental taxation, and current forecasts need to be reconsidered.

### Airport level forecast

We conclude this chapter with a forecast on airport level, taking the German airport Frankfurt (FRA) as an example. The fixed effect was adjusted by inserting a dummy for FRA whilst assigning MUC (Airport Munich) as a placeholder to avoid perfect collinearity, keeping everything else constant from model (1).^9^ We thereby isolate the FRA coefficient with 1.96 (standard error 0.05) being highly significant. While interpretation is not straightforward (see Mummolo and Peterson 2018 for a discussion), it shows that FRA performs above-average globally regarding freight and that the airport infrastructure leads to an additional 2 % freight growth on average. Keeping all airport characteristics constant, we can create a forecast for FRA under the premise that there are no infrastructural bottlenecks.^10^ Changes in infrastructure can be considered in a scenario approach, building categorical links to (structurally) similar airports; however, we refrain from additional constraints in this paper. This could be an avenue of future research.

**Fig. 4:**
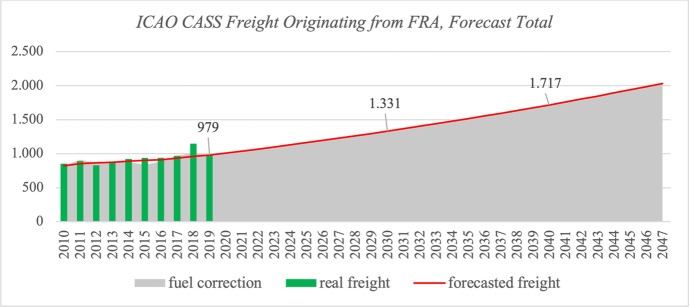
ICAO CASS Freight Originating from FRA, Forecast Total

**Fig. 5:**
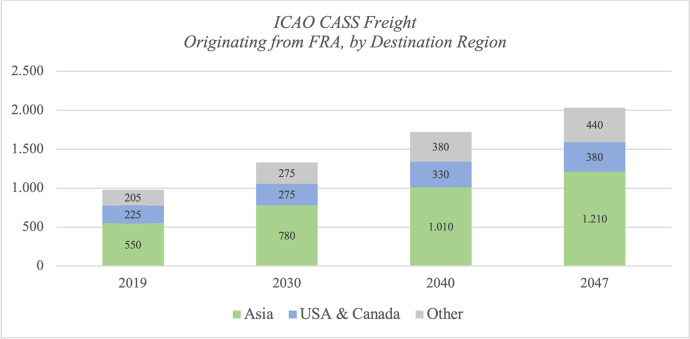
ICAO CASS Freight Originating from FRA, by Destination Region

FRA handled about 980 M ton-kilometers ICAO CASS registered freight in 2019, of which 56 % went to Asia and 23 % to USA & Canada. Intra-Europe freight is mainly handled via trucking, thus we do not find more than 0.5 % of freight originating FRA heading towards other European countries. Figures 4 and 5 illustrate the global forecast for FRA with regional destination splits in absolute numbers.

**Fig. 6:**
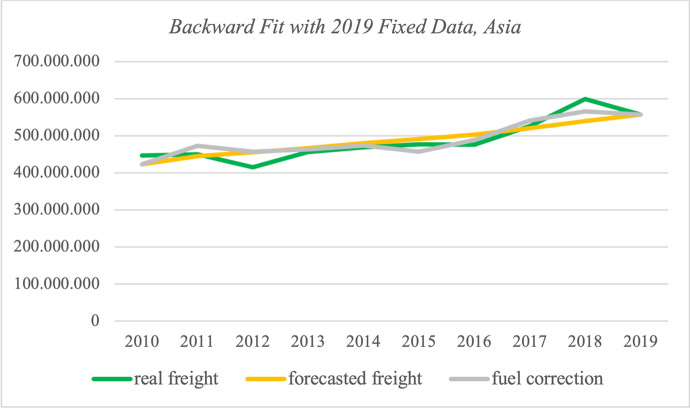
Backward Fit with 2019 Fixed Data, Asia

**Fig. 7:**
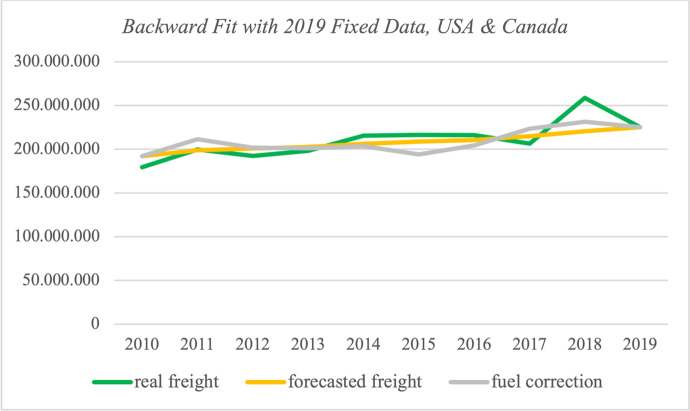
Backward Fit with 2019 Fixed Data, USA & Canada

The back forecast including the fuel correction model is displayed in Figs. 6 and 7 (Appendix) for Asia and USA & Canada, respectively. The growth rate of freight to Asia is about 3.07 % up to 2030 and then slightly decreases until 2047. While the growth rate from FRA to US- and Canadian airports is initially lower, it is forecast to accelerate over time; an overview is given in table 3.

**Table 3:** Average annual growth rates of ICAO CASS freight originating from FRA

	2020 - 2030	2031 - 2040	2041 - 2047
Asia	3,07%	2,65%	2,63%
USA & Canada	1,82%	1,87%	1,95%
Total	2,83%	2,58%	2,43%

Concluding the Frankfurt airport case study, we note that freight is likely to double within the next 30 years and the importance of Asia will increase substantially; it will play a leading role with 60 % of total freight going to Asia in 2047, in which Southeast Asian destination airports will be the major drivers. The share of freight to North American airports is expected to be constant around 20 %, and the role of European airports is rather negligible for FRA.

## Conclusion

The reliability of forecast models in the aviation sector is an important factor for industry and finance likewise. Expanding airports and fleets usually is a cost and time intensive process, and in order to maintain constant efficiency in the market, accurate anticipation of future demand and structural changes is attempted. As we describe in Chapter 2 most air cargo forecast center around models provided by Boeing (constantly being updated; 2020 the latest) and base on theories of gravitation. In Chapter 3 we describe how aviation air freight models can be adapted to meet the state of the art in gravity econometrics (Baier 2020) and programming (Stammann et al. 2016). Airport-dyadic air freight data provided by ICAO CASS is utilized in airport fixed effects ppml models with gravity control variables, standing as robust time invariant (constant) model with GDP per capita as main explanatory (time variant) variable. This simplification makes the model of choice an ideal forecast tool when interpolating GDP per capita prognosis data provided by IHS Markit, as we show in Chapter 4. In addition, by utilizing data on airport level in contrast to country level, the model presented can specify forecasts for single countries, regions and detailed airports – in case sufficient data points are available.

Expanding the level of detail provides an answer for the sign of the distance variable as well, which is controversially discussed in literature until recently; we track negative interactions to a sample selection problem (large airports only) and note that distance generally has no significant impact in cargo aviation gravity. Airport fixed effects models are however found to be limited in a dyadic fixed effects approach often seen in trade literature, as overfitting occurs. Oil prices which are found to have significant impact in previous literature are as well not confirmed in the underlying study – kerosene prices on the other hand are found to remain their significant effect on cargo even in the presence of strong constant models.

Presenting our econometric results and corresponding short-, mid- and long-term forecasts, we are very close to the numbers published by Boeing (2020) short- and midterm, but a little lower the farther we proceed to the future. While we agree with the global average growth of about 4,1 % per year up until 2030, our findings indicate slightly lower growth rates than usually found in literature, with 3,6 % global annual growth 2031–2040 and 3,2 % global annual growth 2041–2047. We explain those deviations by a) different model specifications and b) varying input data sources. On region- and country level deviations increasingly spread due to the methodological nature of approaches.

The prominence of models including airport (fixed) effects shall be highlighted as a concluding remark of this study. As global cargo aviation is likely do double within the next 20 years, this will result in an increasingly complex worldwide transportation network. Small airports which are barely considered today will drastically rise in importance, intergovernmental aviation cooperation is likely to increase especially in the context of environmental economics and policies, and new aviation technologies (from regional air mobility to long distance supersonic) will additionally add complexity to routes. It is thus necessary to track the changes those effects cause on a more detailed airport level in order to anticipate future traffic flow and evasion, which is on the other hand essential for policy makers and the economy to ensure a smooth transition to the aviation sector of the future.

## Data Availability

Not applicable.

## Appendix

**Table 4:** Estimation results for airport-dyadic FE (7) in comparison to airport FE (1); in (7) the airport FE are nested in 181.821 dyadic FE.

	(1)	(7)
log(dist)	-0.009 (0.013)	
log(gdp_cap_o)	0.768*** (0.130)	0.874*** (0.060)
log(gdp_cap_d)	0.525*** (0.111)	0.805*** (0.060)
contig	-0.521 *** (0.052)	
comlang_off	0.303*** (0.022)	
colony	0.665*** (0.024)	
comcol	0.176*** (0.042)	
n	1002201	1001468
l	[1233, 3186, 10]	[181821, 10]
pseudo-R^2^	0,7502	0,9658

Standard errors in parentheses

*** p<0.01, ** p<0.05, * p<0.1

## Footnotes

1 Constant 2010, if not further specified.

2 For robustness checks we decided as well to estimate our model (1) without zeroes and (2) only freight values above 50,000 ton-kilometers.

3 The goodness of fit will only reach a satisfying level if we select an airport with enough data points over the ten-year time frame; thus this interpretation should only be applied to larger and/or more popular freight airports and has to be discussed in regards with regional airport economic situation and constraints.

4 Increasing distance provides time- and cost arguments especially for high quality goods to prefer air transport rather than alternative shipping methods. For a literature review on distance in aviation gravity, see Hazledine (2017) for gobal discussion and Wang et al. (2018) for a China case study.

5 Estimates from OECD’s ”Scenarios for the World Economy to 2060” predict global GDP per capita growth of +3.4 % 2018 to 2030 and +2.4 % 2030 to 2060 (OECD 2018).

6 Country and region-specific forecasts in freight aviation is possible not only with regards to the rest of the world, but with specific other regions and single countries as well; i. e. we can for example generate not only a European- Asian forecast, but a more specialized German-Chinese one.

7 Considering all other parameters – including oil or kerosene prices – to remain constant.

8 Technical measurement for global warming, more precise than greenhouse emissions.

9 While this theoretically changes the fixed effects setting to a minimal extent, we do not observe a change of independent variables coefficients up to their third decimal place; the impact is therefore negligible.

10 See Gelhausen et al. (2019) for possible airport capacity constraints and how to estimate them; note that capacity constraints for freight can differ from those for passengers due to possible night time operation and other effects that level the capacity situation.
